# Gut microbiome responds compositionally and functionally to the seasonal diet variations in wild gibbons

**DOI:** 10.1038/s41522-023-00388-2

**Published:** 2023-04-21

**Authors:** Qi Li, Han-Lan Fei, Zhen-Hao Luo, Shao-Ming Gao, Pan-Deng Wang, Li-Ying Lan, Xin-Feng Zhao, Li-Nan Huang, Peng-Fei Fan

**Affiliations:** 1 School of Life Sciences, Sun Yat-sen University, 510275 Guangzhou, P.R. China; 2 College of Life Science, China West Normal University, 637002 Nanchong, P.R. China; 3 School of Ecology, Shenzhen Campus of Sun Yat-sen University, 518107 Shenzhen, P.R. China; 4 School of Life Sciences, South China Normal University, 510631 Guangzhou, P.R. China

**Keywords:** Microbial ecology, Metagenomics

## Abstract

Wild animals may encounter multiple challenges especially food shortage and altered diet composition in their suboptimal ranges. Yet, how the gut microbiome responds to dietary changes remains poorly understood. Prior studies on wild animal microbiomes have typically leaned upon relatively coarse dietary records and individually unresolved fecal samples. Here, we conducted a longitudinal study integrating 514 time-series individually recognized fecal samples with parallel fine-grained dietary data from two Skywalker hoolock gibbon (*Hoolock tianxing*) groups populating high-altitude mountainous forests in western Yunnan Province, China. 16S rRNA gene amplicon sequencing showed a remarkable seasonal fluctuation in the gibbons’ gut microbial community structure both across individuals and between the social groups, especially driven by the relative abundances of *Lanchnospiraceae* and *Oscillospiraceae* associated with fluctuating consumption of leaf. Metagenomic functional profiling revealed that diverse metabolisms associated with cellulose degradation and short-chain fatty acids (SCFAs) production were enriched in the high-leaf periods possibly to compensate for energy intake. Genome-resolved metagenomics further enabled the resolving metabolic capacities associated with carbohydrate breakdown among community members which exhibited a high degree of functional redundancy. Our results highlight a taxonomically and functionally sensitive gut microbiome actively responding to the seasonally shifting diet, facilitating the survival and reproduction of the endangered gibbon species in their suboptimal habitats.

## Introduction

Human activities significantly affected the survival and reproduction of wild animals^1–4^. In particular, human activities may lead to the shrinking back of wildlife habitats, with many of the populations being constrained in the margins of their original natural ranges^5–7^. In these suboptimal habitats, wild animals face more challenges for survival, including especially food shortage and low-temperature conditions. To cope with these challenges, wild animals may evolve to have behavioral^8,9^ and physiological adaptations including dietary alterations^10^. Importantly, as the digestive system could not catch up to the fast-changing environment, the rapid response of gut microbiota and associated metabolic activities may represent a key mechanism enabling the adaptation of their hosts to suboptimal habitats^11,12^. While recent surveys have suggested that plasticity in gut microbiota may provide dietary and metabolic flexibility to animals^13–15^, longitudinal studies were lacking to systematically investigate how gut microbiome responds to seasonally fluctuating diets for wild animals in their new, non-native ranges.

The investigation of temporal gut microbiome dynamics in wild animals faced multiple difficulties, especially in habituation and individual recognization of animals, long-term monitoring of dietary composition, and collection of fresh fecal samples resolved at the individual level. These aspects were even more challenging when studying tree-dwelling animals in natural settings. Here, we overcame these challenges by adopting a pioneering strategy in which full-day dietary data were recorded and parallel fresh feces were collected for six habituated and individually recognizable wild skywalker hoolock gibbons the newly described and endangered species *Hoolock tianxing*^16,17^, from two family groups for over a year. As typical frugivorous animals originally mainly residing in the tropics, skywalker hoolock gibbons have less developed cecum and relatively round molar crowns that are not adapted to ground and digest fibrous plant materials (e.g., leaves)^16,18^. However, as affected by human activities, the distribution area of wild gibbons has been shrinking back, with some of the remaining populations restricted in high-altitude mountainous forests in western Yunnan Province, China, and eastern Myanmar, which represent the northernmost margin of global gibbon species distribution^16^.

Consequently, these gibbons experienced drastic seasonal fluctuations in dietary composition and were forced to shift to a diet mainly of leaves when fruits were not available during the coldest months of the year^19^. This feature renders them an ideal target for exploring the potential linkage and underlying mechanisms between seasonal diet variations and gut microbial communities of wild animals. We generated an exceptionally massive longitudinal dataset, containing 16S rRNA gene amplicon and metagenomic sequences, to comprehensively investigate the potential adaptive strategies of the gut microbiome to the seasonally fluctuating food resources in two gibbon groups. Our analyses revealed dynamic microbiomes sensitive to drastic diet changes, stressing the individual-specific responses of the microbial community at both the taxonomic and metabolic functional levels. Such insights are crucial for understanding how these wild animals survive and adapt to new and stressful environments.

## Results

### The gibbon gut microbiota profile

**Fig. 1:**
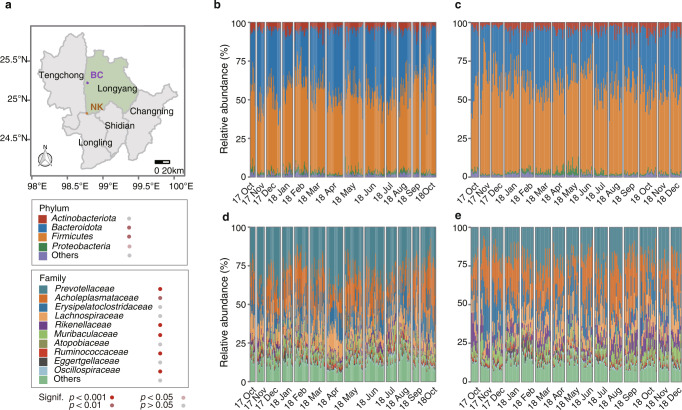
Microbiome composition of 514 gibbon fecal samples. **a** Home ranges of two social groups Nankang (orange, NK) and Banchang (purple, BC) during sample collection. **b**–**e** Relative abundances of phyla (**b** NK; **c** BC) families (**d** NK; **e** BC). Samples were sorted by the sampling time. Each column represented one sample and samples from different months were separated by the blank space. Significance was calculated by the Wilcoxon rank-sum test with samples classified by social groups.

To investigate the potential longitudinal relationship between diet and gut microbiota, we collected dietary data and 514 fresh fecal samples from six individual gibbons (two social groups) over the course of 15 months. Deep 16S rRNA gene V4 amplicon sequencing identified a total of 6750 zero-radius operational taxonomic units (ZOTUs), which were assigned to 943 genera and 42 phyla. 299 ZOTUs (4.6% of the total) were present in at least 90% of the samples, constituting the “core microbiota” of the gibbon gut microbial community (Supplementary Table 1). At the phylum level, the overwhelming majority (70–90%; Fig. 1b and c) of microbes were affiliated with the *Firmicutes* and *Bacteroidota*, with the remainder largely belonging to the *Actinobacteriota* and *Proteobacteria*. At the family level (Fig. 1d and e), the most abundant families included *Prevotellaceae* (27.2 ± 9.9%), *Acholeplasmataceae* (20.0 ± 10.2%), *Erysipelatoclostridiaceae* (13.5 ± 6.5%), and *Lachnospiraceae* (11.1 ± 4.3%). Strikingly, nearly all *Acholeplasmataceae* sequences were assigned to a single ZOTU (ZOTU1), accounting for 19.8% of the total community. Additionally, the most abundant archaeal species (from the phylum *Crenarchaeota*) exhibited a high level of prevalence (detected in all samples), although at a low relative abundance.

### Influence of social group on the microbiota structure

While the overall taxonomic composition of the gut microbiota in both social groups was generally similar, relative abundances of some specific taxa (phylum, family, and genus levels) showed significant differences (Fig. 1 and Supplementary Fig. 1, Wilcoxon rank-sum test, *p* < 0.05). Notably, alpha diversity indexes (Shannon diversity, evenness, observed richness, and Faith’s phylogenetic diversity) were significantly higher in the NK group (Wilcoxon rank-sum test, *p* < 0.001; Supplementary Fig. 2a–d), indicating a more diversified microbiota. The clear separation of gut microbiota by social groups was confirmed by the Bray–Curtis dissimilatory (Supplementary Fig. 2e, ANOSIM; *R*^2^ = 0.67, *p* = 0.001).

SIMPER (similarity percentages procedure) analysis was conducted to identify the specific taxa that contributed to the differentiation of microbial communities in the two gibbon groups (Supplementary Table 2). Characteristic ZOTUs presented at an explicitly higher relative abundance in the NK group included ZOTU5 (family *Prevotellaceae*, with a contribution of 3.4%), ZOTU3 (*Rikenellaceae*, 1.8%), and ZOTU7 (*Lachnospiraceae*, 1.4%), whereas other ZOTUs (including the *Anaeroplasma* ZOTU1 with the highest abundance) were apparently more abundant in the BC gibbons.

### Covariation of diet and gut microbiota across social groups and individuals

**Fig. 2:**
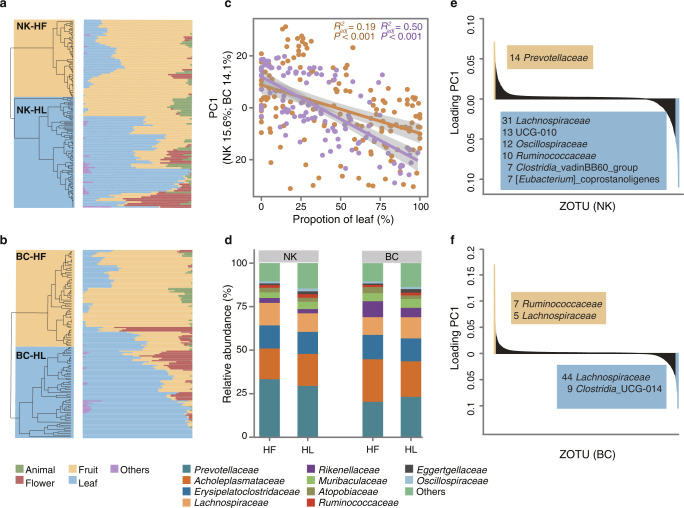
Covariation of diet and gut microbiota across the social groups. **a** and **b** Hierarchical clustering with the dietary composition of the social groups (**a** NK; **b** BC). Detailed information was provided in Supplementary Table 7. **c** The correlation between the first principal component (PC1) of between-sample dissimilarity and the proportion of leaf in the diet with colors representing different subjects (orange, NK; purple, BC). The *p*-values were obtained from linear regression analysis. **d** Gut microbial composition of NK and BC social groups in the high-fruit and high-leaf periods (the dominant families were shown). **e** and **f** Loading PC1 scores of each ZOTU within social groups (**e**, NK; **f**, BC) with a loading score of >0.05 and <0.05 highlighted.

**Fig. 3:**
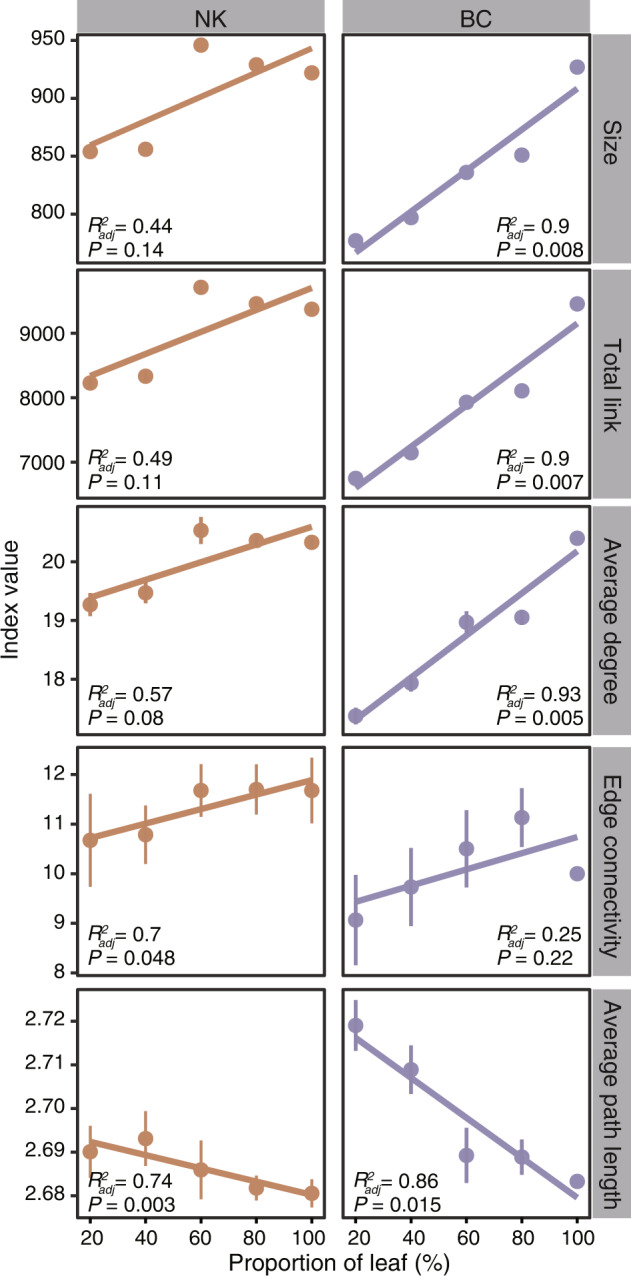
The relationship between the five properties (Size, Total link, Average degree, Edge connectivity, and Average path length) of gut microbiome co-occurrence network and the diet. The points and error bars on the panels represented mean ± standard deviation after subsampling 999 times. Colors denoted different social groups (orange, NK; purple, BC). The adjusted *R*^2^ values for linear regression were presented. The *p*-values were obtained from linear regression analysis.

Fine-grained observational dietary data were applied to investigate how seasonal diet variation may affect gibbon gut microbiota. Hierarchical cluster analysis showed a clear separation of dietary composition into the high-fruit (HF) period and high-leaf (HL) periods (Fig. 2a and b). Notably, a consistent response of gut microbiota to dietary changes across social groups was observed albeit with significant differences in microbial community structure (Fig. 2). Specifically, Shannon diversity and evenness were significantly increased with the proportion of leaves and decreased with that of fruits (Supplementary Fig. 3a–d, *p* < 0.001, Linear regression analysis) in two gibbon groups, while the significance for the observed richness and Faith’s phylogenetic diversity was only detected in the NK group (Supplementary Fig. 3e–h). Besides, microbial community dissimilarity increased with dietary dissimilarity within each social group (Supplementary Fig. 4). Meanwhile, the first principal component of beta diversity which was largely related to diet composition, accounted for 14.0% and 15.6% of the variation in the two social groups (Fig. 2c). All ZOTUs that loaded negatively on PC1 (more abundant during the HL periods) were assigned as *Lachnospiraceae* in both gibbon groups (Fig. 2e and f). In contrast, the ZOTUs that loaded positively on PC1 (more abundant during the HF periods) belonged to the family *Prevotellaceae* in the NK group, and primarily the families *Ruminococcaceas* and *Lachnospiraceae* in the BC group (Fig. 2e and f). SPIEC-EASI co-occurrence networks were further constructed to explore how diet impacted species interactions and the structure of the gut microbial community. The results showed that the gut microbial networks varied considerably over diet. Both gibbon groups exhibited similar increases in the size of networks with more nodes and links along the amount of fiber consumed, albeit only statistically significant in the BC group (Fig. 3). Meanwhile, increasing average degree and edge connectivity were also associated with increased fiber consumption, indicating that the microbial networks became more complex and connective during periods of high fiber availability. Additionally, average path length showed a significantly decreasing trend from the fruit-dominated diet to the leaf-dominated diet for both social groups.

**Fig. 4:**
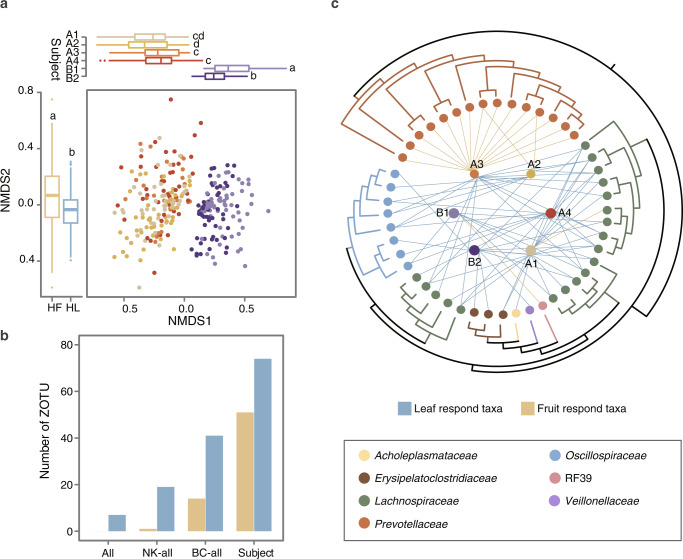
Covariation of diet and gut microbiota across the individuals. **a** NMDS (center panel) of the Bray–Curtis dissimilarity based on the microbial community composition. Different colors corresponded to different gibbon individuals in the top panel. Boxplots (top panel) indicated the distribution of each individual along the first axis of NMDS. The boxplots on the left panel depicted different diet types according to their gut microbial community placement on the second axis of NMDS. Different letters represented significant differences (ANOVA followed by Duncan’s test). **b** The number of the diet-specific responsive ZOTUs. Colors indicated the types of responsive ZOTUs (yellow, responding to fruit; blue, responding to leaf). **c** Within-individual correlations between diet and the relative abundances of ZOTUs (Spearman’s correlation *r* > 0.6; FDR-corrected *p*-value < 0.01). The colored circles in the middle represented different gibbon individuals. The tree outside was obtained by 16S rRNA gene sequences, and the colors represented diverse microbial families. The lines with different colors indicated the types of responses.

The separation of microbial communities of the six gibbons along the first NMDS axis (Fig. 4a) indicated gut microbiota varied widely across individuals. To gain deeper insight into this pattern, Procrustes analysis was performed to explore microbiota variation and dietary variation across individuals. The results showed that an individual’s daily dietary composition generally corresponded with its microbiota composition (Supplementary Fig. 5, Monte Carlo *p*-value = 0.001). Consistently, a significant positive correlation between the degree of seasonal turnover in diet and microbiota composition across individuals was observed. Overall, the variance in microbiota turnover could be primarily (49–62% of variance) explained by diet turnover (Supplementary Fig. 6). Furthermore, the Spearman correlation was conducted to determine which taxa varied monotonically with an increasing proportion of leaves or fruits in diet across individuals (Supplementary Table 3). The result showed that ZOTUs with significant correlations (false discovery rate < 0.05) with diet types (fruit or leaf) were relatively personalized, indicating that the diet-sensitive ZOTUs were not always conserved across individuals (Fig. 4b; but see Supplementary Fig. 7 for the seven examples with common response). Notably, the ZOTUs of leaf response had an obvious richness and phylogenetic diversity than those of fruit response (Fig. 4b and c) and, when adopting more strict thresholds (Spearman’s correlation *r* > 0.6; FDR-corrected *p*-value < 0.01), they were always conserved at the family level. Specifically, *Lachnospiraceae* and *Oscillospiraceae* represented the main leaf response taxa, while microbes of fruit response were almost exclusively affiliated with *Prevotellaceae*.

### Functional responses of gut microbiota to dietary changes

To obtain a deeper insight into the observed association between gut microbiota and diet changes, we generated 3.27 T of metagenomic sequence data from the 99 fecal samples of gibbons A2 and B2 (representing the NK and BC groups, respectively, mean: 27.7 ± 2.9 Gb). A total of 3,906,912 putative protein-coding genes were predicted, of which 39.5% could be annotated in the KEGG database (Supplementary Fig. 8a and b) and 2.2% annotated as carbohydrate-active enzymes (CAZymes, Supplementary Fig. 8c and d). More than half (51.9%) of the genes could not be assigned to known functions.

**Fig. 5:**
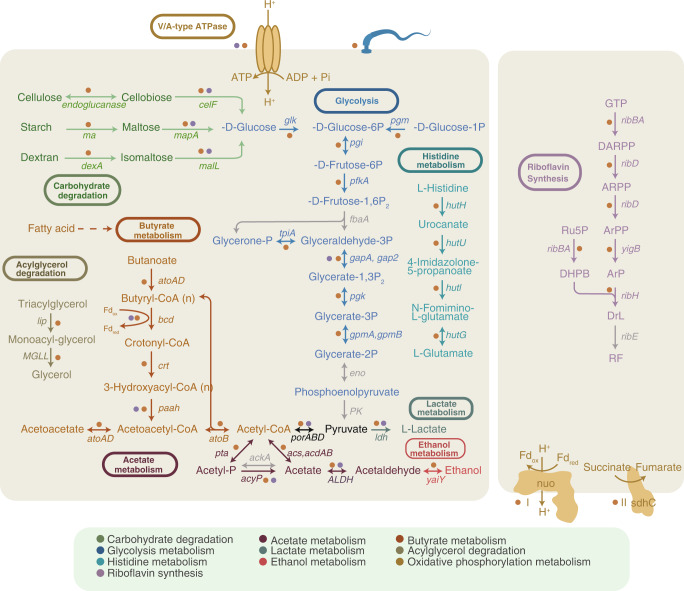
Overview of the functional profiles depicting the pathways enriched in different diet periods with colors differentiating gibbon subjects (orange, A2; purple, B2). The diagrams showed metabolic pathways enriched during high-leaf (left) and during high-fruit (right). The colors of different solid lines corresponded to significantly differential metabolic pathways (Wilcoxon rank-sum test, and FDR-corrected *p*-value < 0.05). The genes with no significant difference between the two diets were marked with gray lines. The detailed information on the genes was summarized in Supplementary Table 9.

The clustering patterns of the metagenomic functional profiles of the two gibbons demonstrated a clear covariation between gut microbial community functions and dietary types (Supplementary Fig. 8e and f). The core KO categories were further examined to identify the significantly represented pathways associated with the different diet periods (Supplementary Fig. 9). Compared with the high-fruit (HF) periods, a considerably wider range of KEGG pathways, including membrane transport, carbohydrate metabolism-, and energy metabolism-related KO categories were enriched during high-leaf (HL) periods in both gibbon individuals (Supplementary Fig. 9; *p* < 0.05, Fisher’s exact test). Specifically, enrichment in specific pathways involved in complex carbohydrates degradation, including cellulolytic (endoglucanase and 6-phospho-beta-glucosidase) and dextran-lytic (dextranase and oligo-1,6-glucosidase) enzymes were clearly evidenced, suggesting enhanced capacities for nutrient digestion and utilization. Additionally, the increased relative abundances of cellular energy metabolisms (especially V/A type ATPase, Fig. 5) during the HL periods may provide additional energy to host in nutrient-depleted conditions. Similar trends of genes for volatile SCFAs production (e.g., butyrate, acetate, and lactate) were observed during the HL periods, particularly in A2 (Fig. 5). Notably, some other pathways were found to be uniquely enhanced in the A2 gibbon; these included the Embden–Meyerhof–Parnas (EMP) glycolysis pathway, histidine metabolism, acylglycerol degradation, and cell motility during the HL periods and the riboflavin synthesis pathway, Complex I and II during HF periods (Fig. 5).

**Fig. 6:**
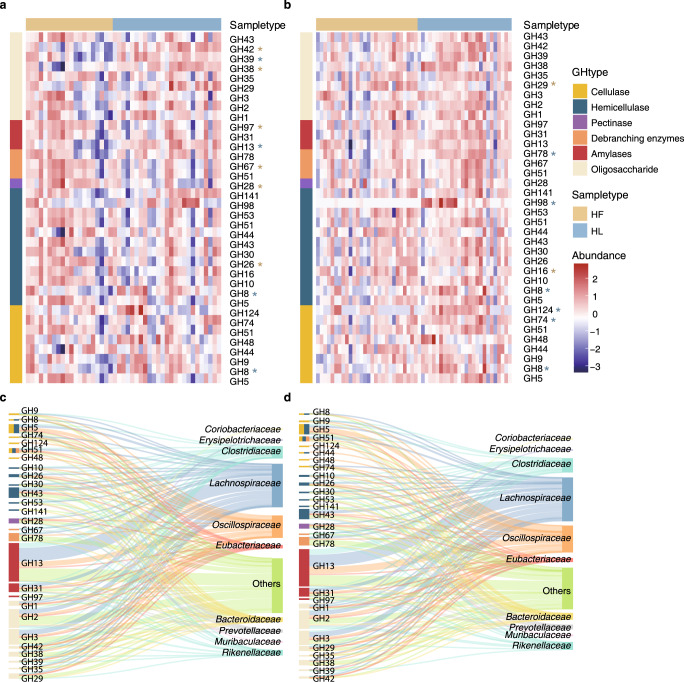
CAZymes profile adapted to polysaccharide metabolism. **a** and **b** Heatmap of the CAZymes profile in the gibbon gut metagenomes (**a** A2; **b** B2). CAZymes associated with cellulase, hemicellulase, pectinase, debranching enzymes, amylases, and oligosaccharide degradation were presented. CAZymes families with significant enrichment (Wilcoxon rank-sum test, and FDR-corrected *p*-value < 0.05) in the leaf- and fruit-dominated periods were marked. The standardized relative abundances of each CAZymes family were shown. **c** and **d** Sankey diagram describing the distribution of the top 10 microbial families associated with all selected CAZymes families ranked by the number of genes in gibbons A2 (**c**) and B2 (**d**). CAZymes were represented by different colors according to functional types and microbes were colored according to the associated families. The heights of the rectangles indicated the numbers of the CAZymes (left) and microbial species (right). The detailed information on the genes was summarized in Supplementary Table 10.

The predicted protein-coding genes were mapped to the CAZymes database to further resolve the complex carbohydrate utilization in the gut microbiome. Nearly half of the genes with CAZymes annotation were assigned as glycoside hydrolases (GHs, 46.8 ± 2.1%), including the digestion of oligosaccharides (25.3 ± 2.9%), hemicellulose (16.6 ± 2.7%), starch (12.8 ± 1.7%), cellulose (5.5 ± 0.9%), and pectin (4.3 ± 1.8%). Again, apparent separation of CAZymes profiles along the diets was observed for both gibbon individuals (Supplementary Fig. 8g and h). Notably, genes related to cellulose and hemicellulose degradation enzymes were enriched during HL periods either in B2 (e.g., GH8, GH74, GH98, GH124) or in A2 (GH8) (Fig. 6a and b). Meanwhile, the amylase-encoding GH13 was found to be significantly over-represented in A2 during the same periods (Fig. 6c and d). Taxonomic assignment showed that these HL-associated CAZymes genes mainly belonged to the *Lachnospiraceae*, *Oscillospiraceae*, *Eubacteriaceae*, and *Prevotellaceae*, indicating critical roles of these taxa in fiber digestion and utilization in the gibbon gut ecosystem.

Given the individual-dependent responses of the gut microbial populations to the diet, an additional set of samples for the other four gibbon subjects within the typical dietary periods were further selected for metagenomic sequencing. Metagenomic analysis of the 38 representative fecal samples revealed generally similar clustering patterns of overall community function and CAZymes profiles shaped by diet variation (Supplementary Fig. 10) and identified additional KOs (especially those associated with pathways in membrane transport, signal transduction, carbohydrate metabolism, and energy metabolisms, Supplementary Fig. 10c and e) and CAZymes (Supplementary Fig. 10d) enriched in HL periods.

### Resolving carbohydrate degradation and fermentation metabolism at the MAGs level

To further resolve the connection between community members and functional capacities, 13,388 prokaryotic genomes were assembled from 138 fecal metagenomes. The metagenome-assembled genomes (MAGs) were then dereplicated into 569 representative genomes at the species level. Compared with the high-fruit periods, a higher richness and phylogenetic diversity of MAGs were enriched during the high-leaf periods, resembling patterns of the 16S rRNA-based analysis (Supplementary Table 4). We then focused on these differentially enriched MAGs to explore the capability of interconnected microbial functional guilds to convert plant polysaccharides into soluble sugars. For this purpose, the genomes were assigned to the different trophic levels along the carbon food chain inferred from the linkages to specific substrates, including (1) complex carbohydrate polysaccharides, (2) sugar utilization, and (3) sugar fermentation.

**Fig. 7:**
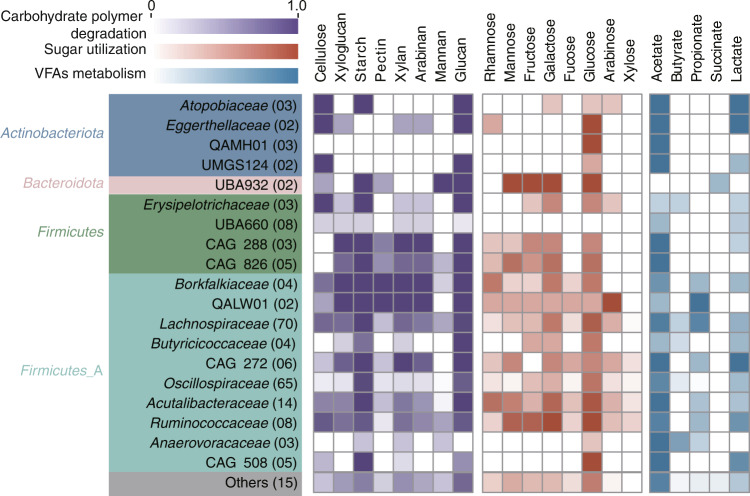
Metabolic reconstruction of MAGs enriched in high-leaf diets. Heatmap indicated the proportions of MAGs with the abilities for major polysaccharides degradation, sugar utilization, and fermentation. The MAGs were summarized into family-level taxonomic clades based on the phylogeny inferred by GTDB-Tk. Functional profiles were reconstructed inferred from the presence of the key marker genes within the metabolic pathway. Detailed information was provided in Supplementary Table 5.

A degradation potential of a large number of complex carbohydrate polysaccharides (including cellulose, hemicellulose, and starch) was identified in the reconstructed *Lachnospiraceae*, *Acutalibacteraceae*, *Borkfalkiaceae*, and *Ruminococcaceae* MAGs (Fig. 7 and Supplementary Fig. 11). The cellulose decomposition ability of the majority of these taxa could be inferred by the detection of cellulase families GH5, GH8, GH9, and GH45, of which GH5 was the most common (Supplementary Table 4). Notably, GH48 was only detected in a *Ruminococcaceae* MAG (B055_bin_86), indicating a potential for degradating cellulose while also possessing cohesion and dockerin domains associated with cellulosomes (Supplementary Table 5). In addition, the vital roles of these enriched taxa in starch and glucan biodegradation could be reflected by the wide detection of amylase and β-glucosidase (Supplementary Table 5). The distribution of the potentials of the next-level substrates, including rhamnose, mannose, fructose, galactose, fucose, and glucose, was similar to that of the complex sugar, stressing a metabolic versatility of gut populations. Notably, the genes for the utilization of a large number of substrates (12 or more) co-occurred in these enriched taxa (Supplementary Fig. 11), which may serve as metabolic hubs providing highly diversified capabilities to cope with polymer degradation in the gibbon gut. Unlike the two trophic levels above, the third one was composed of a limited number of SCFA producers, with the utilization of acetate and lactate contributing the most. The major butyrate and propionate fermenters were assigned to *Lachnospiraceae*, and the potential of succinate fermentation was scatteredly distributed in only four MAGs within the families *Burkholderiaceae*, *Lachnospiraceae*, *Bacteroidaceae*, and UBA932.

## Discussion

To investigate the potential impacts of seasonal dietary variation on gut microbiota, recent works typically collected and analyzed fecal samples left behind by a specific social group of wild animals^14^. Thus, our direct sampling of fresh and individually resolved feces from the wild gibbons, coupled with parallel time series feeding data, represented a major step forward, enabling insights into the gut microbiome and dynamics of this endangered primate species.

By integrating the simultaneously collected fine-grained dietary data, we demonstrated that the gibbon gut microbiome was driven by the shifting dietary modes associated with the seasonal availability of resources. Different subjects responded to the seasonal diet variations in a similar way, despite considerable differences in their gut microbial community structure. Moreover, gibbons with more dissimilar diets also had more dissimilar microbiomes, and the variation of microbiome turnover could be largely explained by dietary turnover within each subject (explainable variation ranging from 49% to 62%, Supplementary Fig. 6), which is significantly higher than those previously reported for large-herbivore species in African^20^. A possible explanation was that we quantified diet and microbiome composition precisely for each gibbon individual, thus eliminating the effect of host lineage on their potential linkages. Notably, elevated microbiota alpha diversity (richness and evenness) and the number of KOs were detected with increased intake of plant fiber, possibly mirroring a need for more diverse microbial populations for a hard-to-digest diet^21,22^. This result was expected since the transformation of complex substrates into end products is typically accomplished through a number of metabolic stages mediated by inter-connected microbial groups^23–25^.

With the functional versatility encoding in many different MAGs, the metabolic repertoires of the gibbon gut microbiome exhibited a high degree of redundancy, which may provide functional plasticity and stability during dietary changes. Specifically, when a given taxon is compromised in suboptimal conditions, the niche could be supplemented by other functionally similar counterparts enabling a buffering against environmental perturbations at the community level^26^. Such a feature has been documented in rumen animals and human microbiomes^27–29^, where phylogenetically diversified functional guilds could maintain overall metabolic activities and growth under a wide range of environmental conditions including dietary fluctuations. The functional redundancy also led to an enhanced species competition stress among the microbial interactions during the high-leaf periods (Supplementary Fig. 12). The crucial role of competition in maintaining microbial species diversity and stabilizing microbial community composition has been stressed^30^, which reflects the potential of recovering the original state from internal or external disturbances. This stability may be essential to host health, as the maintenance of beneficial microbial symbionts and their associated functions in the gut is ensured over time^26,31–33^.

Recent investigations have suggested that some microbial taxa or metabolic pathways may respond to specific dietary compounds in humans^34^. In the present study, we observed highly positive correlations between increased leaf intake and relative abundances of certain bacterial taxa (e.g., *Lachnospiraceae* and *Oscillospira*), indicating a pivotal role of these microbes in fiber degradation in the gibbon gut. This finding was reasonable since *Lachnospiraceae* spp. are the main cellulolytic taxa in the mammalian gut^13,35–37^, producing short-chain fatty acids in the cellulolytic processes, and bacteria from the family *Oscillospira* have been found highly prevalent in cattle and sheep rumen^38^, and their relative abundance was associated with seasonal diet variation in the wild wood mice^39^. The metagenome-derived functional profiles showed that *Lachnospiraceae* and *Oscillospira* harbored various functional CAZymes involved in cellulose and hemicellulose degradation (Fig. 6c and d), supporting these taxa as keystone species for utilizing high-fiber food in the wild gibbons. On the other hand, *Prevotella*, which is capable of digesting non-cellulosic polysaccharides, pectin, and soluble sugars as energy sources^40,41^, was markedly enriched in the gibbon gut microbiota during periods of high-fruit availability. Similar results have been previously reported in western lowland gorillas^14,42^ and geladas^13^.

The role of gut microbiota in the host adaptation to dietary variation could also be reflected in the synergistic actions in community function profiles. Our metagenomic analysis revealed that diverse genes involved in cellulose and hemicellulose breakdown (e.g., endoglucanase, GH8, and GH124) were significantly enriched in HL periods, indicating efficient metabolic capabilities for the sequential conversion of these depolymerized polysaccharides into glucose. The high functional potential to produce SCFAs (such as butyrate, acetate, and lactate) in the HL metagenomes may imply compensation for energy intake efficiency by microbial fermentation. In support of this, previous studies have demonstrated that volatile SCFAs not only provide a substantial fraction of the daily energy supply for folivorous primates, sheep, and cattle^43–45^ but also are crucial in anti-inflammation and improving immunity^46^. Thus, our findings indicated a flexible functional configuration of the gibbon gut microbiota to obtain energy through fermentation to SCFAs or conversion to glucose suggesting a microbiome adaptive to the utilization of complex cellulose. In comparison, the overall gut microbiota function was more constrained and reflected a diet high in soluble sugars during HF periods (Fig. 5). Notably, our metagenomic analysis uncovered that the riboflavin (vitamin B_2_) synthesis pathway was significantly elevated in the HF periods (Fig. 5 and Supplementary Fig. 10), indicating its important role in the gibbon gut microbial community as riboflavin is critical in cellular metabolism and is involved in the carbohydrate, amino acids, and energy-producing metabolisms^47,48^.

In humans, altering the availability of diet has been found to change the taxonomic composition and functional profiles of the gut microbiome, resulting in intestinal health problems such as obesity and autoimmune disease^49–51^. In this study, we observed an increase in the relative abundance of *Heliobacter* (Supplementary Fig. 13), a potential pathogen genus that infects humans and other vertebrates^52,53^, during the shift from a fruit-dominated diet to a fibrous diet. This result was consistent with prior studies which reported an increase of potentially pathogenic microbes with fruit availability possibly due to more nutritional stresses during fruit scarcity^37,54^. Additionally, we found that two beneficial commensal genera, *Bifidobacterium* and *Lactobacillus*, were also negatively correlated with fruit availability. We anticipated that these genera would provide a balance for the gibbons to maintain healthy gut homeostasis. Understanding the balance between diet transmissions of pathogenic versus beneficial bacteria may thus provide a new approach to monitoring the health of wild animals.

Increasing evidence shows that the properties of co-occurrence networks may reflect interactions between co-existing taxa which could affect the gut microbial community’s response to host/environment-associated variables^55,56^. Furthermore, previous studies have reported resource and food availability as the key factor shaping the social network structures of gut microbiota^57,58^. Our results showed that the gibbon gut microbiome networks responded similarly to the shifting diet in the two social groups, with a stronger impact of diet observed in the BC group. Compared to the microbial network associated with a fruit-rich diet, the fiber-rich diet network was larger in size and relatively complex, indicating more mutualistic interactions, guilds, or niches sharing among microorganisms^59,60^. These results suggested that, in order to obtain adequate energy from energy-poor fibrous materials, a more diverse microbial community with a more complex network of metabolic interdependencies to ferment the low nutrients into SCFAs is required for the wild gibbons. In principle, these complex microbial social networks may provide the gibbon hosts with dietary flexibility, allowing maximized energy extraction and thus improved fitness.

**Fig. 8:**
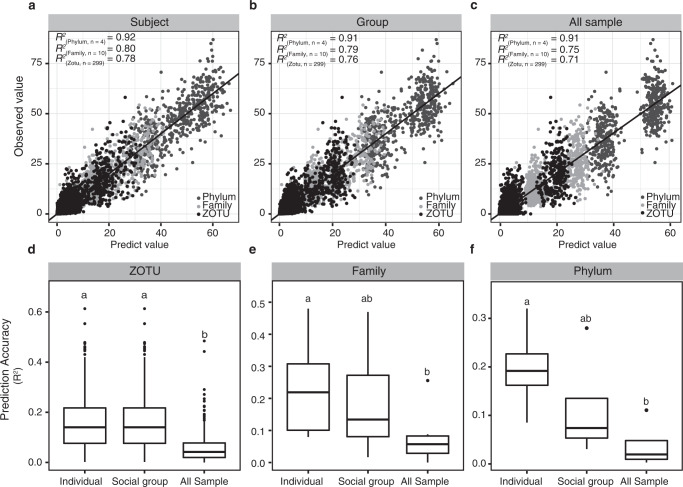
Prediction performance between different biotic levels in different sampling scales. The comparison of cross-validation results (**a**–**c**) and prediction accuracies (**d**–**f**) between different biotic levels in different sampling scales. **a**–**c** Scatter plots showing the predicted and observed values of relative abundances of different biotic levels inferred from dietary compositions. Colors represented different biotic levels (dark gray: phylum; light gray: family; black: ZOTU). The diagonal line represented perfect prediction (predicted value = observed value). **d**–**f** The accuracy of random forest models in predicting relative abundances in different taxa. Significant differences among different datasets were denoted by lettering (*P* < 0.05, ANOVA followed Duncan’s test). The detailed information was summarized in Supplementary Table 6.

Our massive 16S rRNA gene and metagenomic data sets have revealed dynamic gibbon gut microbiomes sensitive to seasonal dietary changes. Such a community is compositional and functional responses were largely personalized, resulting in a gut microbiota better predicted at the individual level than at the whole sample level (Fig. 8; see also Supplementary Table 6). The comprehensive set of genomes assembled from the metagenomes has further enabled resolving the metabolic capacities associated with carbon degradation among different community members. Future studies are needed to utilize these MAGS to investigate how other important functions are partitioned in the community and how taxa interact, either competitively or cooperatively, in response to the fluctuating diet. Given the critical importance of gut microbiota to wild animals, a comprehensive mechanistic understanding of its temporal dynamics and functional consequences in Skywalker hoolock gibbons will ultimately contribute to the development of effective conservation strategies to help this endangered species adapt to their new, suboptimal habitats.

## Methods

### Study sites and gibbon individuals

Our research was conducted at two sites within Mt. Gaoligon National Nature Reserve, Yunnan, China: Nankang (NK, 24°49′N, 98°46′E) from October 2017 to October 2018, and Banchang (BC, N25°12′, E98°46′) from October 2017 to December 2018 (Fig. 1a). Both habitats are strongly seasonal, with the majority of the precipitation falling between May to October at NK and between June to October at BC. The annual mean temperature at NK was 13.3 °C from October 2010 to September 2011^19^. Among them, the minimum temperature was −2.2 °C in January, and the maximum was 30.9 °C in July. Similarly, the annual mean temperature at BC was 13.0 °C from June 2013 to May 2015^61^, with the lowest temperature −3 °C dropped in December and the highest temperature at 33.6 °C in June. The Skywalker hoolock gibbons in this area have been continuously monitored by our group for over 10 years. Thus, different individuals cloud be identified based on facial characteristics and body scars. In the present study, we focused on four individuals (consisting of an adult male, an adult female, and two juveniles) at NK and two individuals (an adult male and an adult female) at BC. A 10-year-old subadult female from NK died during the sampling period (July 2018).

### Fecal sample collection and feeding behavior observation

To ensure sample quality, fecal samples were collected immediately after defecation (typically less than 5 min). Fecal samples free of soil and litter contamination were carefully and aseptically collected. Samples were placed into 50-ml sterile tubes with 95% ethanol^62^ and then transported to the laboratory, where they were stored at −80 °C prior to subsequent processing.

Diet data resolved at the individual level were collected^8^. In brief, each month we followed the gibbons for on average eight days. Gibbons were located by visiting their sleeping sites of the previous day, listening to their loud calls, and visiting the fruit trees that the gibbons frequented. Once found, gibbon members were tracked until they arrived at their sleeping sites. Through continuous observation, the food species and food type (including fruits, leaves, flowers, animals, and unknown/unidentified diet items) eaten by gibbons were recorded by using two methods (5-min scan and ad libitum)^63^. The observed time gibbons spent on specific food types was used to quantify the proportions of each food type instead of a direct estimation of the amount of food consumed. Hierarchical cluster analysis of dietary compositions was conducted with the average linkage method (hclust argument) based on Bray–Curtis dissimilarity (using vegan) and visualized with ggtree. The detailed information of each sample was summarized in Supplementary Table 7.

### 16S rRNA amplicon sequencing, bioinformatic and statistical analyses

DNA was extracted from the fecal samples using the ALFA-SEQ Stool DNA Kit (Magigene, Guangdong, China) following the manufacturer’s instructions. Subsequently, the V4 hypervariable region of the 16S ribosomal RNA genes was amplified using the primer set F515 (5’-GTGCCAGCMGCCGCGGTAA-3’) and R806 (5’-GGACTACVSGGGTATCTAAT-3’). The resulting PCR products were pooled in equal amounts, and paired-end 250 bp sequencing was carried out on an Illumina NovaSeq 6000 platform (Illumina, San Diego, CA). The generated 16S rRNA gene sequences were pre-processed (including assembling paired-end reads, trimming primer, and quality. filtering) using the USEARCH (v.10.0.240)^64^ The resulting sequences were denoised into Zero-radius Operational Taxonomy Units (ZOTUs) at the 100% similarity threshold. Putative chimeras were eliminated using UNOISE2^65^. Taxonomy classification of each ZOTU was performed in QIIME 2 (v.2019.10)^66^ with the QIIME2 feature classifier plugin^67^ and the SILVA (v.138) sequence database^68^. ZOTUs that were classified as chloroplasts or mitochondria were removed. The taxonomic classifications of ZOTUs were summarized in Supplementary Table 8. To allow comparison on an equal basis, data were rarefied to 83,000 reads per sample (*n* = 514) for the downstream comparison analyses. Then, we averaged ZOTUs abundance for all samples on the same day within the same diet and rarefied to 82,600 reads per sample for the subsequent analyses.

All statistical analyses were implemented in R (v.4.0.2). Alpha diversity (i.e., observed richness, Shannon diversity, and evenness diversity) was calculated using the Microbiome package. The statistical significance of differences in alpha diversity and taxonomic composition between the two social groups was determined using the non-parametric Wilcoxon rank-sum test. The between-samples difference was evaluated with Aitchison distance and the principal component analysis (PCA) in “prcomp” function, enabling the projection of each sample and the variable loadings of ZOTU onto individual principal components (PCs). Regression analysis was used to test the association between diet and microbiome. SIMPER analysis was applied to identify taxa that primarily contributed to the observed differences in microbiota between two social groups^69,70^. Multivariate relationships among the microbiota of gibbon social groups and individuals were visualized with principal coordinates analysis (PCoA) and non-metric multidimensional scaling (NMDS) (vegan 2.5-6). The significant variance between groups was evaluated using the anosim function from vegan package with 999 permutations. Procrustes analysis was performed to determine the level of association between microbiome composition and diet composition (vegan 2.5-6). Estimated *p*-values were based on the Monte Carlo permutation tests (999 permutations). Spearman correlations were conducted using the Hmisc package to assess the relationships between microbiome composition and diet composition, and *p*-values were adjusted for multiple comparisons using the FDR method^71^. Hierarchical cluster analysis was conducted based on Bray–Curtis dissimilarity using the “hclust” argument and visualized with heatmap.2 function.

### Co-occurrence network analysis

Network analysis was performed using the sparse inverse covariance estimation with ecological association inference (SPIEC-EASI) method (method = ‘mb’, lambda.min.ratio = 0.001, nlambda = 20, pulsar.params = list (rep.num = 50, ncores = 20)^72^. Samples from the two social groups were divided into five clusters (0–20%, 20–40%, 40–60%, 60–80%, and 80–100%) based on the proportion of leaves in the diet. Networks were built for each cluster separately, and only ZOTUs detected in at least half of the samples were used for network construction to avoid accidental associations caused by low-abundant ZOTUs^73^. The number of samples varies between different clusters, which may affect the comparison of co-occurrence networks between different clusters^74^. In order to eliminate this effect, we re-sampled the cluster with the smallest sample number 999 times randomly. The network topological parameters (network size, total links, average degree, average path length, and edge connectivity) were calculated in the R package igraph^75^.

### Prediction of microbial community composition

By applying random forest modeling^76^, the microbial community composition in response to the diet across different individuals, social groups, and all samples was predicted. Each dataset was split into two parts: 70% as the training data and 30% as the validation data. In this study, we selected different taxonomic levels including phylum (top four phyla), family (top ten families), and core ZOTUs for further analyses. A shuffled dataset for null model testing was generated by randomizing the labels related to the real training set. Then, random forest modelings were performed with the observed and shuffled datasets as input by the R package “randomForest” (random Forest function, with 1000 trees trained). The accuracy of the random forest model was evaluated by mean absolute error (MAE), which was calculated as follows^77^:

$${{{\mathrm{MAE}}}} = \frac{1}{m}\mathop {\sum }\limits_{i = 1}^m \left| {y_i - {{{f}}}\left( {x_i} \right)} \right|$$

where *y*_*i*_ is the observed value of the response variable, *f*(*x*_*i*_) is the predicted value. The difference between predicted models and null models was compared by the Wilcoxon rank-sum test. The differences in diverse groups were compared with one-way ANOVA and Duncan’s multiple tests in R package “agricolae” (aov and duncan.test function). The performance of the models was evaluated by the coefficient of determination (*R*^2^) of the linear regression models of the observed and predicted values.

### Metagenomic assembly and gene profile construction

Metagenomic sequencing was performed on a total of 137 samples with detailed dietary records from six individuals (average metagenome size 28.8 ± 3.2 Gb). The libraries were constructed and sequenced on Illumina NovaSeq 6000 platform (paired-end 150 bp reads). Raw reads were quality filtered to remove low-quality reads and adapter sequences using fastp (v.0.21.0) with default parameters^78^, and host contamination was removed by mapping the read to the host genome with Bowtie2 (v.2.2.9)^79^. Clean reads assembly was performed with Megahit (v.1.2.9) using kmers set of 21,29,39,59,79,99,119,141^80^. Open-reading-frames were predicted from the contigs longer than 500 bp using Prodigal (v.2.6.3)^81^. A non-redundant gene catalog was constructed by clustering all genes using CD-HIT (v.4.8.1)^82^ at 95% nucleic acid identity. The high-quality reads from each sample were mapped to the gene catalogs using Bowtie2 with default parameters. Normalized coverage was estimated based on contigs length. The reads were calculated by multiplying the average number of reads across all libraries and then dividing by the number of reads in each library.

### Functional annotation and taxonomic classification

KEGG Orthologs (KOs) were identified by aligning genes against the KOFAM database using the hmmsearch (v.3.3.2, *e*-value <1e−5). To perform enrichment analysis, we first used the Wilcoxon rank-sum test to test for each gene difference across the two dietary stages. Then, the total number of enriched KOs within the specific pathway was summarized and Fisher exact test was performed to detect the enriched diet-specific pathway. CAZymes were annotated against the dbCAN2 database^83^ (available July 2021) using the hmmsearch (*e*-value < 1e−5) tools of HMMER. The significantly different CAZymes between the two dietary stages were identified using the Wilcoxon rank-sum test. All *p*-values were corrected by the Benjamini–Hochberg false discovery rate (FDR). The taxonomic classification of predicted CAZyme genes was aligned against the NCBI-NR database with Diamond (v.2.0.6) in blastp option (*e*-value < 1e−10) at thresholds of identity >90% and alignment coverage >70%, which the best result with the highest average similarity was defined as the final annotation of the gene. SankeyMATIC (http://sankeymatic.com/) was used to visualize the relationships between bacteria host and CAZymes.

### Metagenome-assembled genomes reconstruct and analysis

High-quality reads were mapped to the scaffolds (≥1000 bp) separately to obtain the coverage of the scaffolds in the respective samples using bowtie v.2.4.5^79^. These scaffolds were used for binning using MetaBAT v.2.12.1^84^ with default parameters, considering the GC content, tetranucleotide frequencies, and abundance profiles of the scaffolds. All MAGs were manually curated using RefineM v.0.1.2^85^. Completeness and contamination of the MAGs were assessed with CheckM v.1.2.0^86^. Only MAGs with genome completeness ≥50% and contamination <10% after manual curation were chosen for further analysis. The metagenome-assembled genomes (MAGs) were then clustered at an estimated species level (ANI ≥ 95%) with dRep v.3.2.2^87^ using the parameters ‘-comp 50 -con 10 -pa 0.9 -sa 0.95 -cm larger -nc 0.3’, resulting in 569 MAGs. The taxonomic classification of the MAGs was conducted with GTDB-Tk v.2.1.1^88^. Open reading frames (ORFs) of these MAGs were determined using Prodigal v.2.6.3^81^ with the “-p single” option. The functional annotations were obtained as mentioned above. The abundance of the MAGs was computed as the fraction of metagenomics reads that were assigned to the scaffolds of each MAGs. Genome-scale metabolic models for the representative genomes were reconstructed using CarveMe v.1.5.0^89^, which were further applied to evaluate complementary and competition indices between pairs of MAGs using the R package RevEcoR v.0.99.3^90^.

## Supplementary information

Supplementary Figure

Supplementary Table 1

Supplementary Table 2

Supplementary Table 3

Supplementary Table 4

Supplementary Table 5

Supplementary Table 6

Supplementary Table 7

Supplementary Table 8

Supplementary Table 9

Supplementary Table 10

## Data Availability

All 16S rRNA raw sequences and raw shotgun metagenomic sequencing data have been deposited in the National Genomics Data Center, China National Center for Bioinformation or Beijing Institute of Genomics, Chinese Academy of Sciences, under the project number PRJCA012504.

## Code availability

The R scripts and relevant data were provided on GitHub at https://github.com/lettial/Gibbon-gut-microbiome.
