# Supplementary material for: Gut microbiome responds compositionally and functionally to the seasonal diet variations in wild gibbons
Source: NPJ Biofilms Microbiomes. 2023 Apr 21;9:21. doi: 10.1038/s41522-023-00388-2 (PMC10121652; doi:10.1038/s41522-023-00388-2)
Supplement: Supplementary file 1 — Supplementary Figure [file 41522_2023_388_MOESM1_ESM.pdf]

Supplementary Materials for

Gut microbiome responds compositionally and functionally to the seasonal diet variations in wild gibbons

Qi Li[1], Han-Lan Fei[1,2], Zhen-Hao Luo[1], Shao-Ming Gao[1], Pan-Deng Wang[3], Li-Ying Lan[1], Xin-Feng Zhao[4], Li-Nan Huang[1*], Peng-Fei Fan[1*]

***Corresponding author:**

Li-Nan Huang, eseshln@mail.sysu.edu.cn

Peng-Fei Fan, fanpf@mail.sysu.edu.cn

This PDF file includes:

Supplementary Figure 1 to 13

Other Supplementary Materials for this manuscript include the following:

Supplementary Table 1 to 10

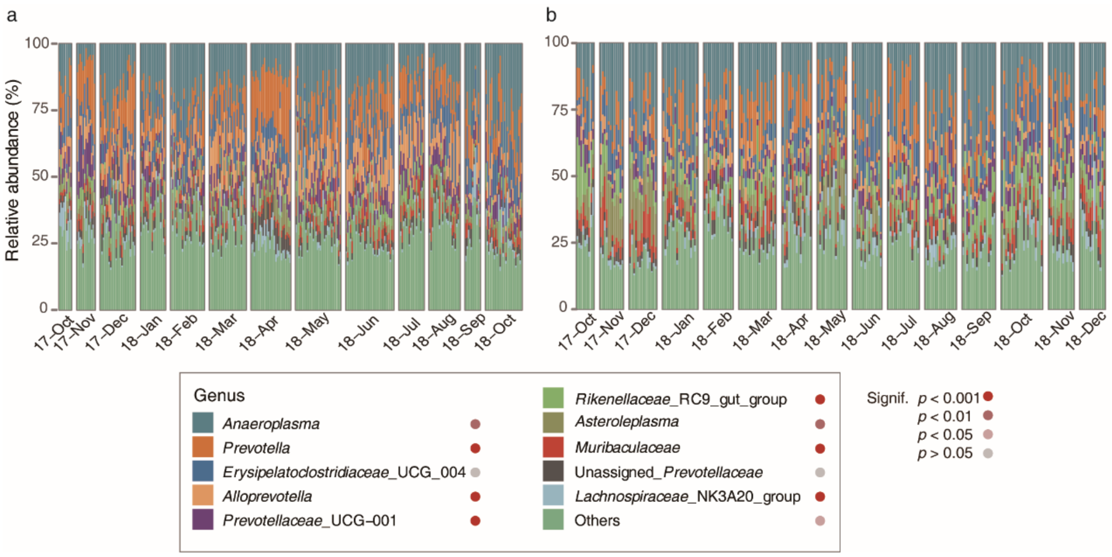

Supplementary Figure 1 | Relative abundances of genera in each fecal microbiome. Samples were sorted by the sampling time (**a**, NK**;** **b**, BC). Each column represented one sample and samples from different months was separated by the blank space. Significance was calculated by the Wilcoxon rank-sum test.

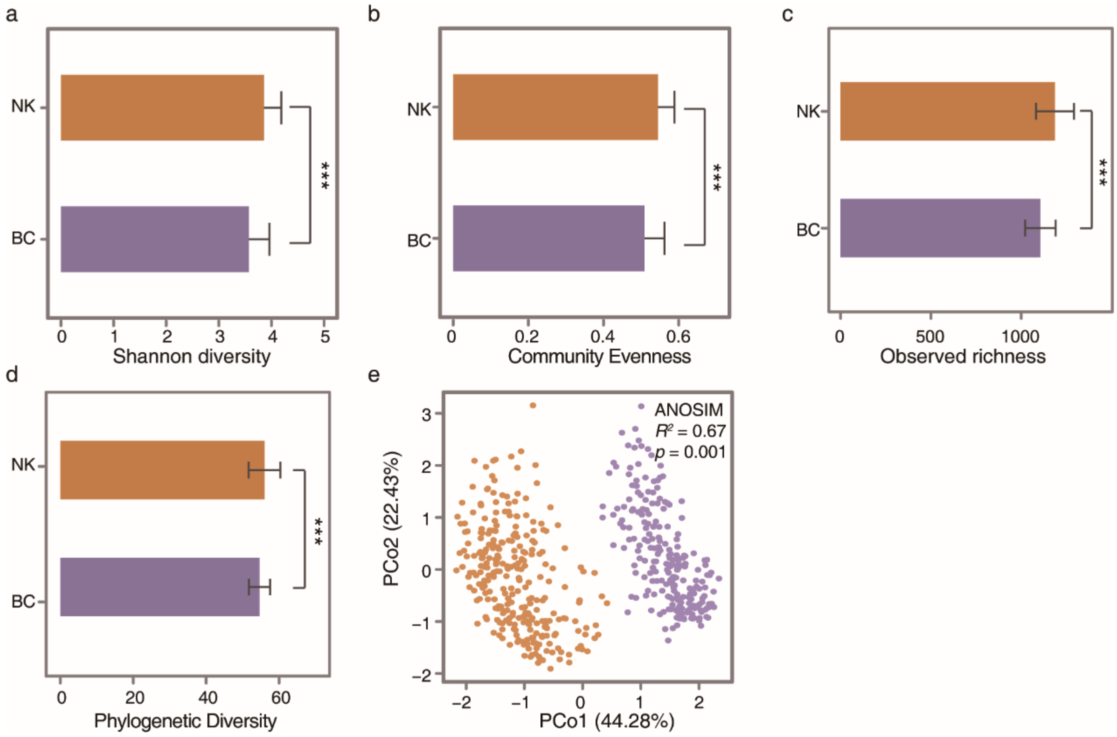

Supplementary Figure 2 | Social group structures the gibbon gut microbiome. The significance of differences in the alpha diversity of (**a**) Shannon, (**b**) evenness, (**c**) observed richness, and (**d**) phylogenetic diversity between different social groups were identified by the Wilcoxon rank-sum test. (**e**) Principal coordinate analysis (PCoA) of all gibbon gut microbiome samples based on the Bray-Curtis distance matrix. Analysis of similarity (ANOSIM) statistics considered samples grouped by social groups. Colors denoted different social groups (orange, NK; purple, BC). $*p < 0.05$; $**p < 0.01$; $***p < 0.001$.

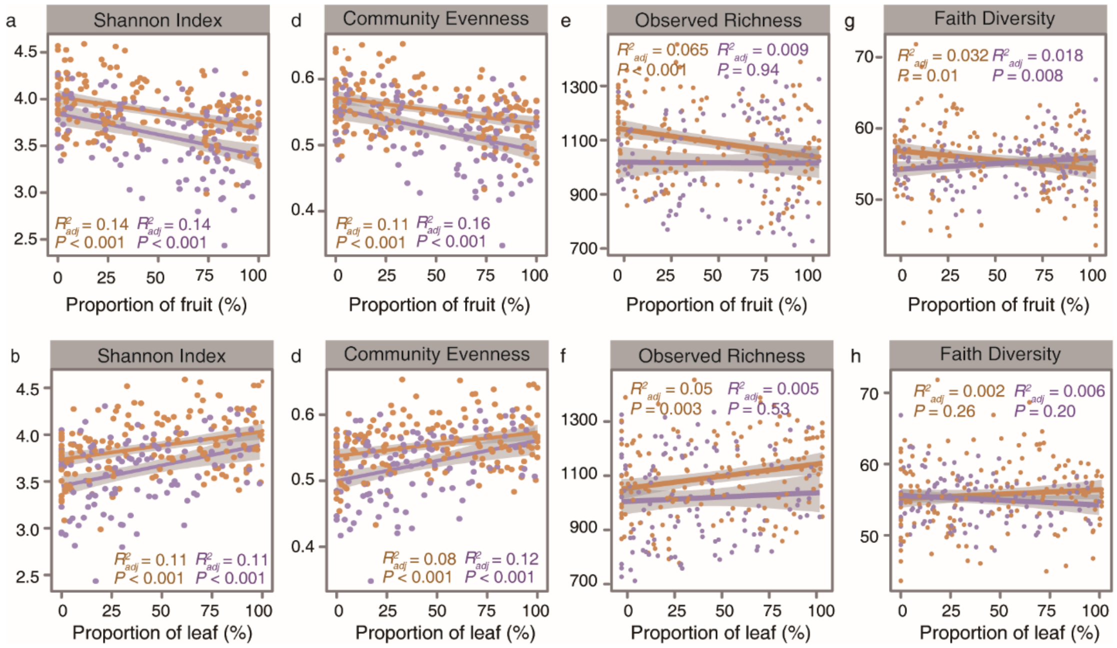

Supplementary Figure 3 | Significant correlations between alpha diversity and diet composition across social groups (orange, NK; purple, BC). The significant correlations between the Shannon Index, Community Evenness, Observed Richness, and Faith Diversity and the proportion of fruits (**a**, **c**, **e**, **g**) and leaf (**b**, **d**, **f**, **h**) were presented. Grey shadow represented the 95% confidence interval. The adjusted R^2 values for linear regression were presented. The *p*-values were obtained from linear regression modeling.

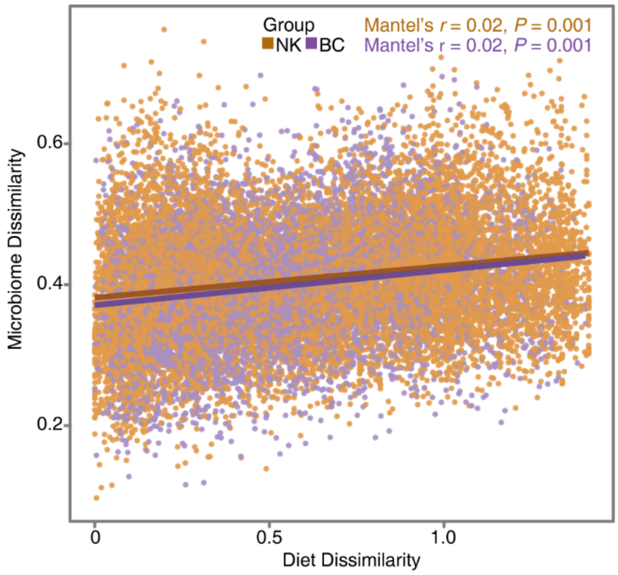

Supplementary Figure 4 | Relationship between dissimilarities of microbiome structure and the diet was evaluated by Mantel's correlation. Color-coded best-fit lines and *p*-values were obtained by Mantel's test.

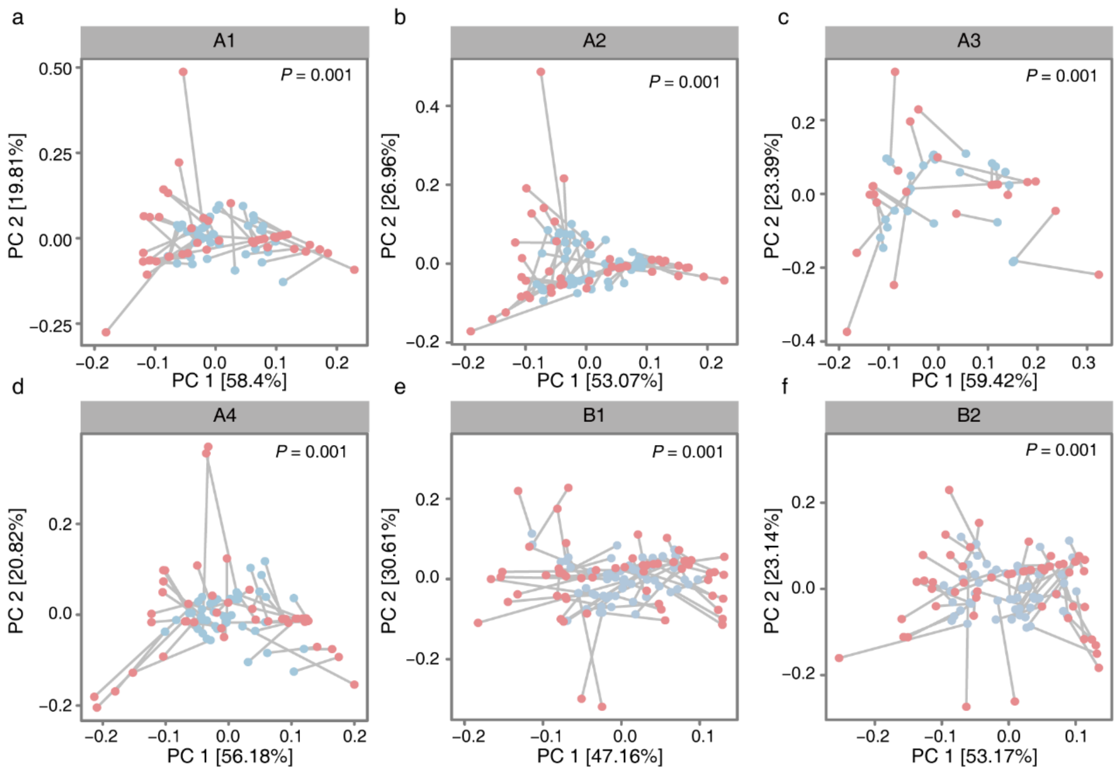

Supplementary Figure 5 | Procrustes analyses to visualize how each individual's seasonal diet and microbiome composition map to each other. (**a-f**) Procrustes rotated the results of separate PCA of diet composition (red circle) and gut microbiota composition (blue circle). The line showed correspondence between diet and microbiome of each sample. The significant dissimilarity between diet and microbiome ordinations was tested by the Monte Carlo *p*-value.

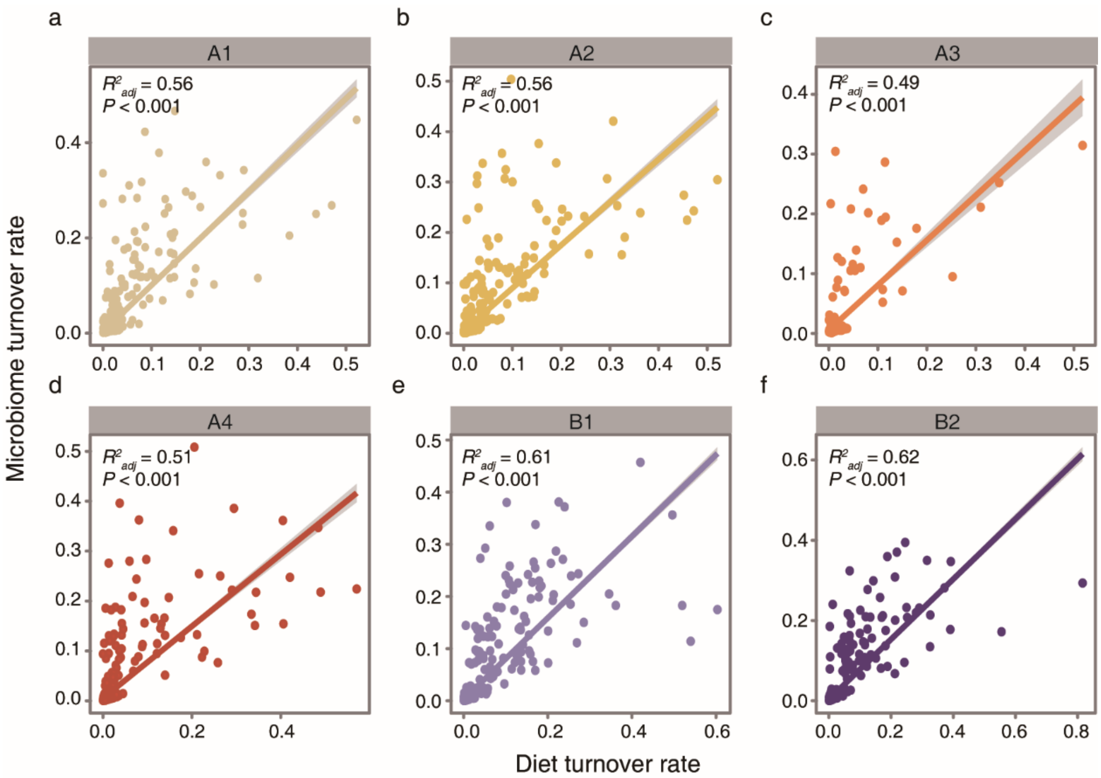

Supplementary Figure 6 | Season turnover of gut microbiota across individuals. (**a-f**) The significant correlations between the seasonal turnover rate in diet and microbiome composition across different gibbon individuals were shown. Different colors corresponded to individuals in the top panel. The adjusted R^2 values for linear regression were presented. The *p*-values were obtained from linear regression modeling.

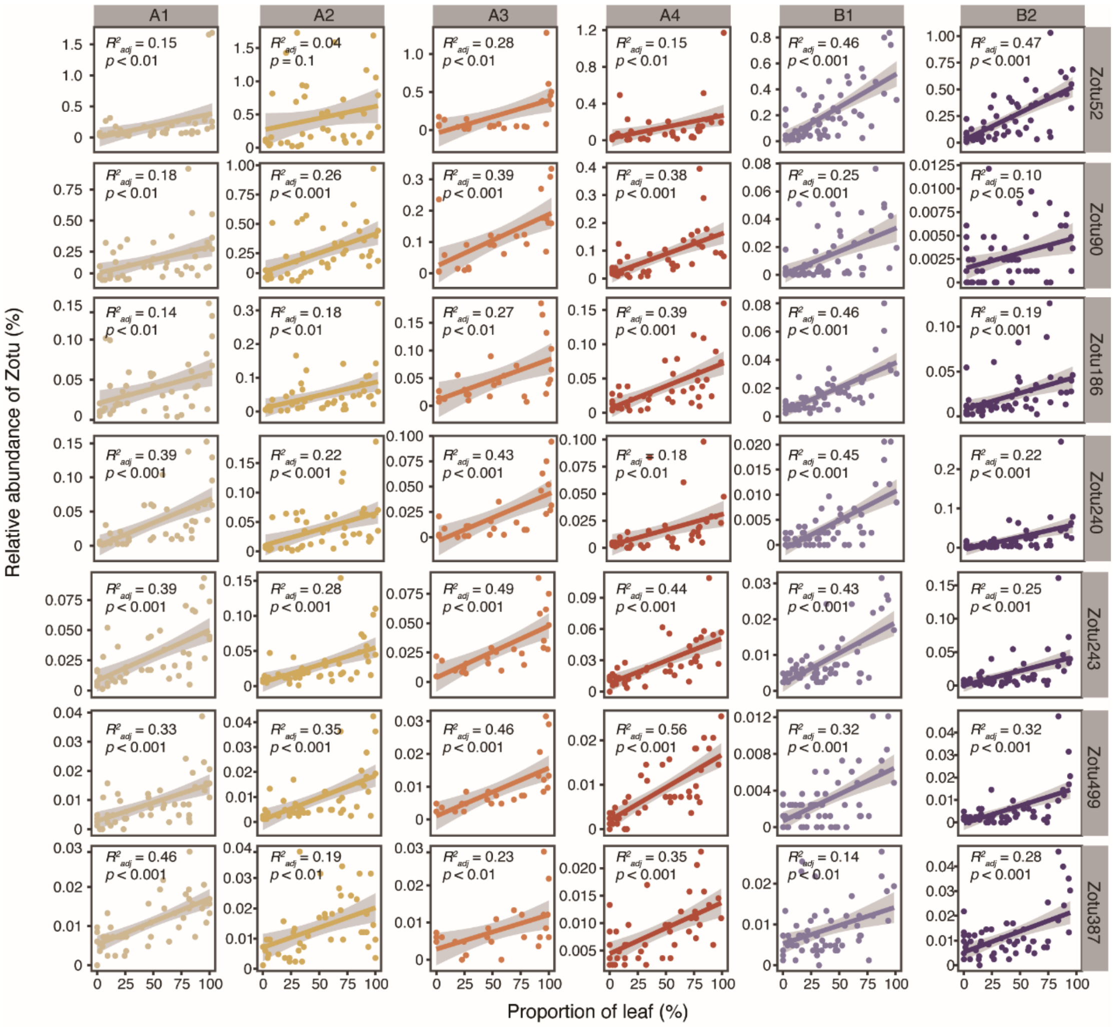

Supplementary Figure 7 | Significant correlations between the relative abundances of seven ZOTUs and the diet with only significant correlations across all individuals were shown. The lines and confidence intervals were obtained from the linear regression. The adjusted R^2 values for linear regression were presented. The *p*-values were obtained from linear regression modeling.

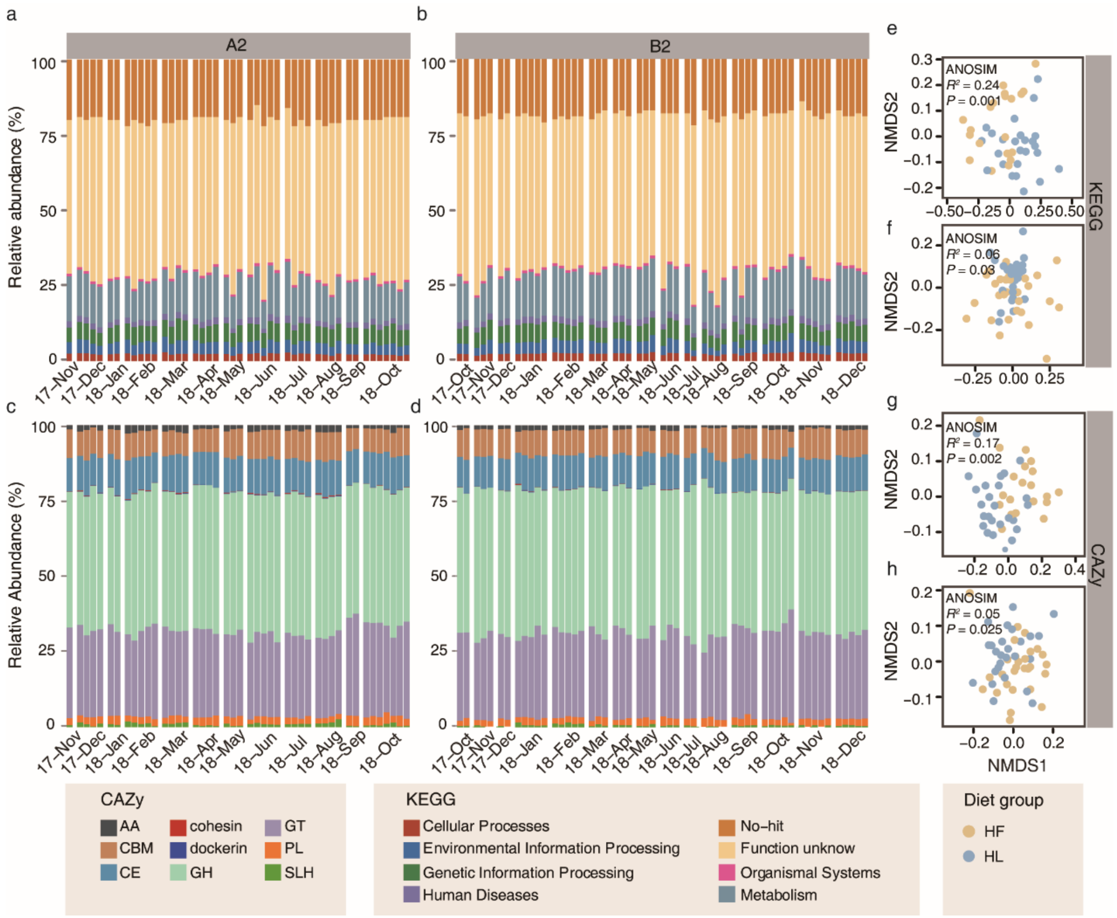

Supplementary Figure 8 | Longitudinal functional profiles of two gibbons. Relative abundance of (**a** and **b**) KO categories and (**c** and **d**) CAZymes families across two gibbons (**a** and **c**, A2; **b** and **d**, B2). NMDS plot of Bray-Curtis dissimilarity in two gibbon individuals (**e** and **g**, A2; **f** and **h**, B2) based on the functional profiles of (**e** and **f**) KO categories and (**g** and **h**) CAZymes families. Analysis of similarity (ANOSIM) statistics considered samples grouped by different diet types. Colors denoted different diet types (yellow, high-fruit; blue, high-leaf).

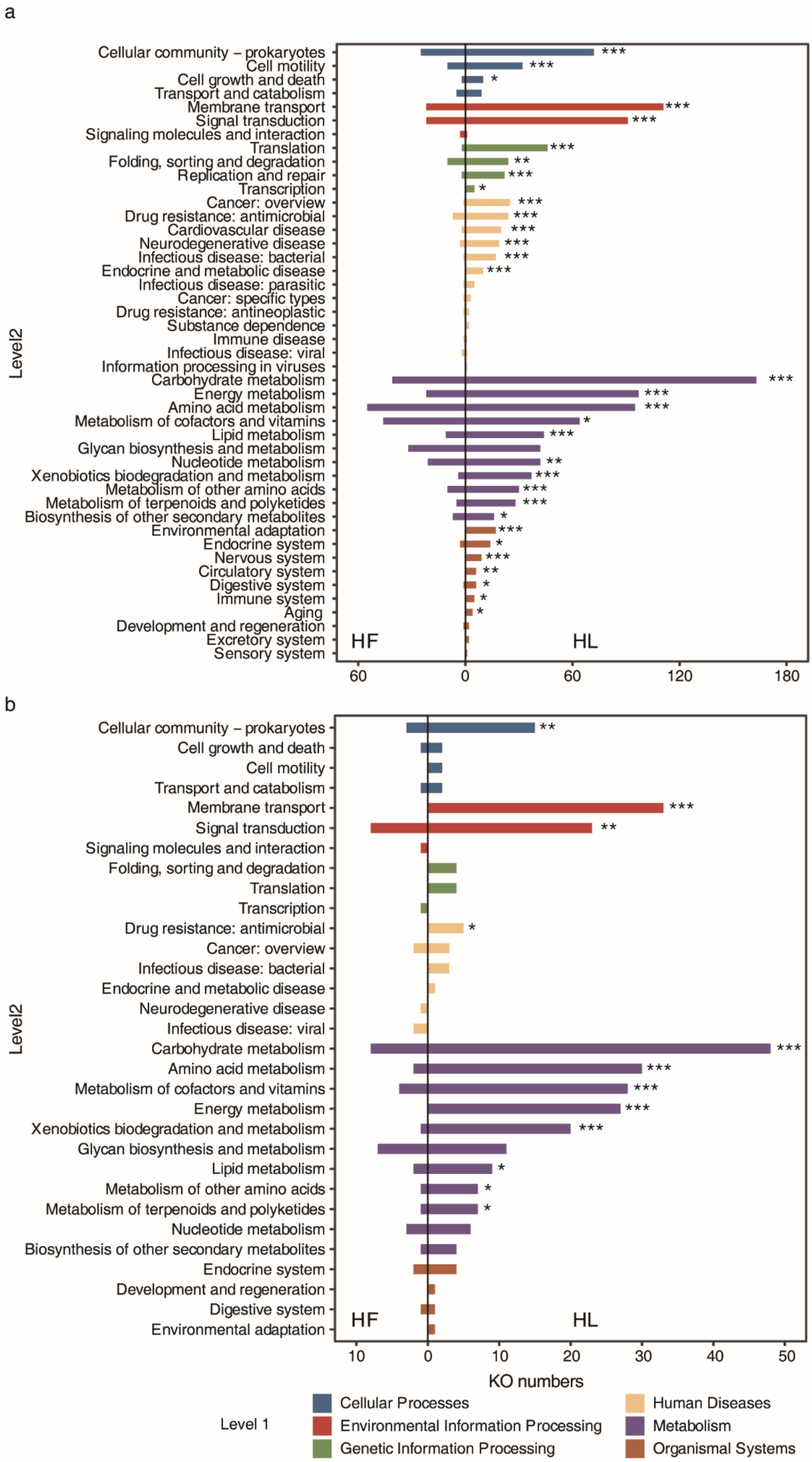

a
b
Level2
Cellular community – prokaryotes
Cell motility
Cell growth and death
Transport and catabolism
Membrane transport
Signal transduction
Signaling molecules and interaction
Translation
Folding, sorting and degradation
Replication and repair
Transcription
Cancer: overview
Drug resistance: antimicrobial
Cardiovascular disease
Neurodegenerative disease
Infectious disease: bacterial
Endocrine and metabolic disease
Infectious disease: parasitic
Cancer: specific types
Drug resistance: antineoplastic
Substance dependence
Immune disease
Infectious disease: viral
Information processing in viruses
Carbohydrate metabolism
Energy metabolism
Amino acid metabolism
Metabolism of cofactors and vitamins
Lipid metabolism
Glycan biosynthesis and metabolism
Nucleotide metabolism
Xenobiotics biodegradation and metabolism
Metabolism of other amino acids
Metabolism of terpenoids and polyketides
Biosynthesis of other secondary metabolites
Environmental adaptation
Endocrine system
Nervous system
Circulatory system
Digestive system
Immune system
Aging
Development and regeneration
Excretory system
Sensory system
HF
HL
60
0
60
120
180
10
0
10
20
30
40
50
KO numbers
Level 1
Cellular Processes
Environmental Information Processing
Genetic Information Processing
Human Diseases
Metabolism
Organismal Systems

Supplementary Figure 9 | Enrichment of the functional profiles of gibbon microbiomes in different diet types. Significant differences of KEGG Orthology (KO) categories (Level 2) between HF and HF diet types in two gibbon individuals (**a**, A2; **b**, B2). The *p*-values generated from two-sided Fisher's exact test (10,000 bootstrap replicates) were indicated as follows: *$p < 0.05$; **$p < 0.01$; ***$p < 0.001$.

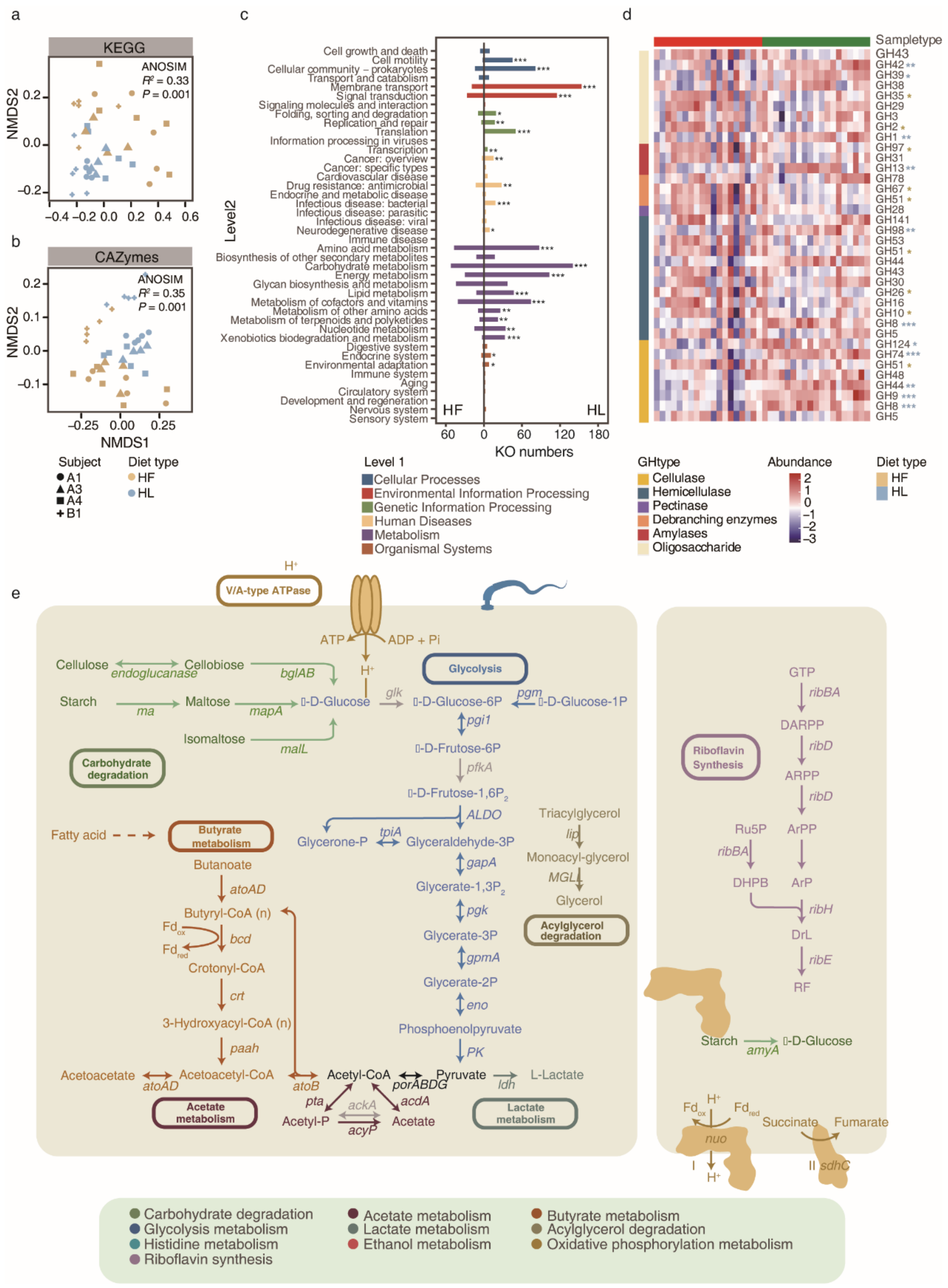

Supplementary Figure 10 | Overview of functional profiles response to different diet types. NMDS plot with Bray-Curtis dissimilarity based on the functional profiles of (**a**) KO categories and (**b**) CAZymes families. Different colors represented samples in distinct dietary types and shapes indicated different subjects. Enrichment of

functional profiles of gibbon microbiomes in different diet types (**c**). The *p* values generated from two-sided Fisher's exact test (10,000 bootstrap replicates) were indicated as follows: **p* < 0.05; ***p* < 0.01; ****p* < 0.001. Heatmap of the CAZymes profile based on the standardized relative abundances of each CAZymes family (**d**). CAZymes associated with cellulase, hemicellulase, pectinase, debranching enzymes, amylases, and oligosaccharide degradation were presented. CAZymes families with significant enrichment in the leaf- and fruit-dominated periods were marked. The detailed information on the genes was summarized in Supplementary Table 7. Overview of functional profiles depicting the pathways enriched in high-leaf (left) and high-fruit (right) periods (**e**). The colors of different solid lines corresponded to different metabolic pathways. The gray lines indicated the absence of pathways. The detailed information on the genes was summarized in Supplementary Table 8.

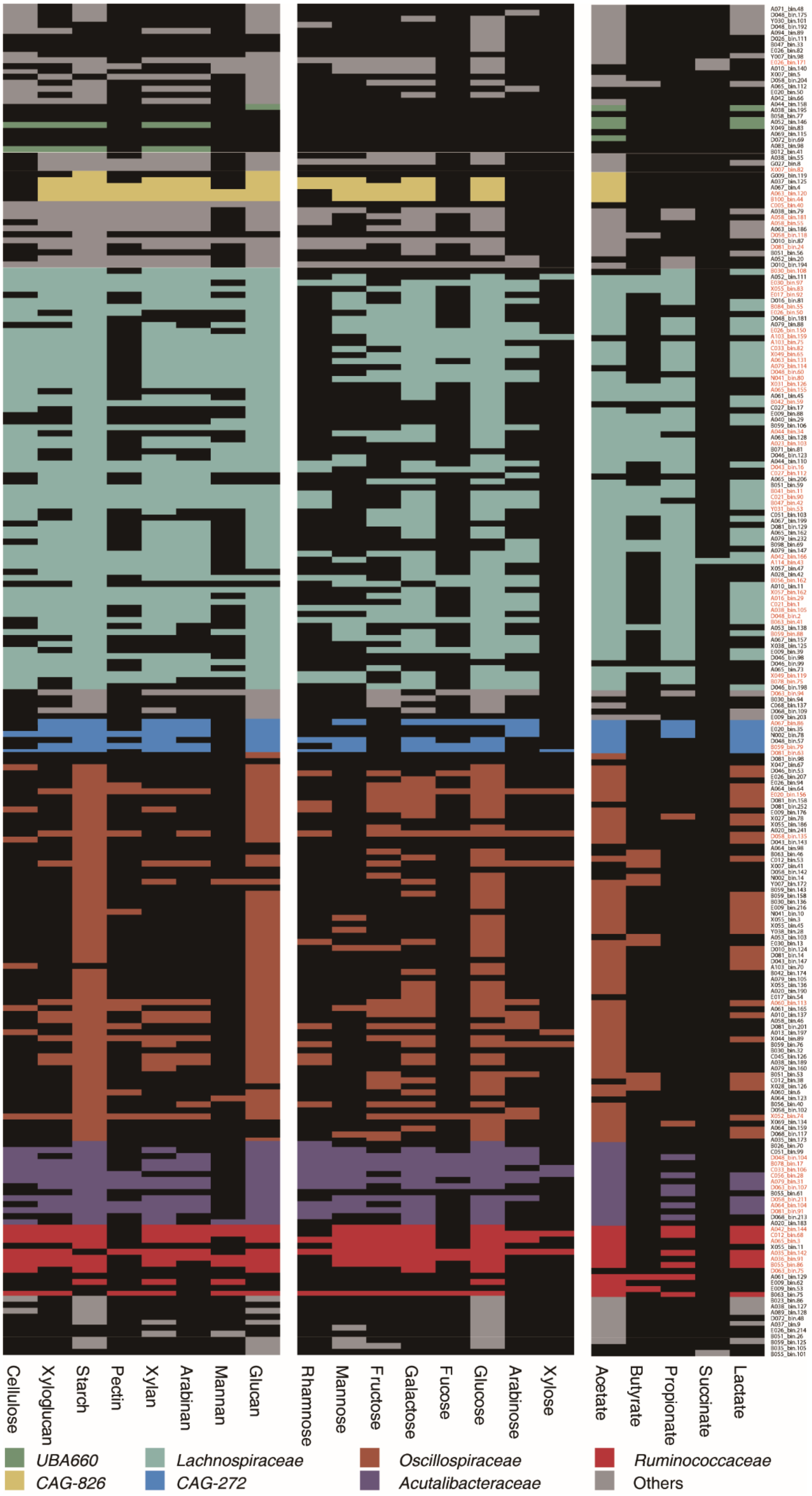
Cellulose
Xyloglucan
Starch
Pectin
Xylan
Arabinan
Mannan
Glucan
Rhamnose
Mannose
Fructose
Galactose
Fucose
Glucose
Arabinose
Xylose
Acetate
Butyrate
Propionate
Succinate
Lactate
UBA660
CAG-826
Lachnospiraceae
CAG-272
Oscillospiraceae
Acutalibacteraceae
Ruminococcaceae
Others

Supplementary Figure 11 | Metabolic reconstruction of each MAGs enriched in high-leaf diets. Heatmap showed the presence or absence of the abilities for major polysaccharides degradation, sugar utilization, and fermentation. The phylogeny (family level) of the MAGs inferred from GTDB-Tk were indicated with different colors. The MAGs with 12 abilities detected (21 considered in total) were labeled in red. The detailed information was provided in Supplementary Table 5.

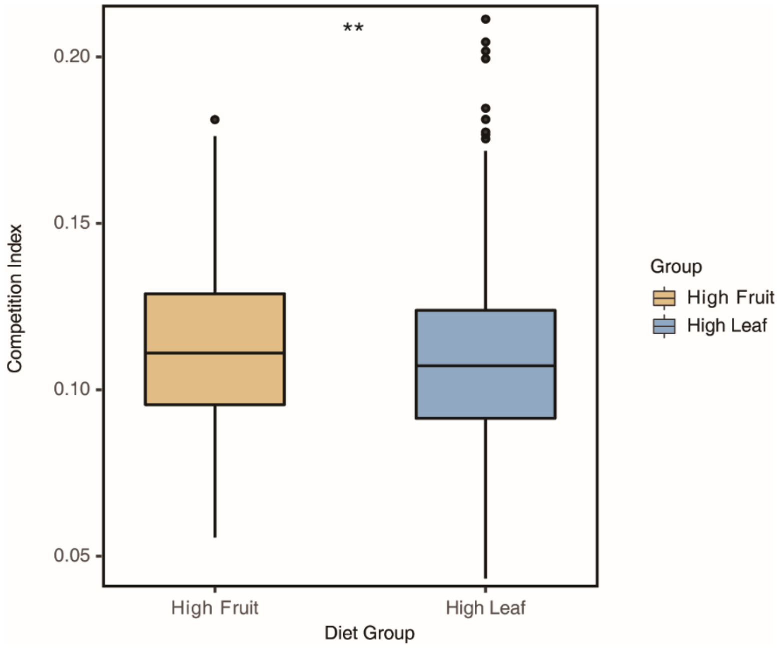

Supplementary Figure 12 | The boxplot showed the average competition index among the MAGs enriched in the high-leaf diet during different periods (yellow: high fruit; blue: high leaf). The metabolic interaction indices were inferred from CarMe and RevEcoR. The *p*-value was obtained from the Wilcoxon rank-sum test. $^*p < 0.05$; $^{**}p < 0.01$; $^{***}p < 0.001$.

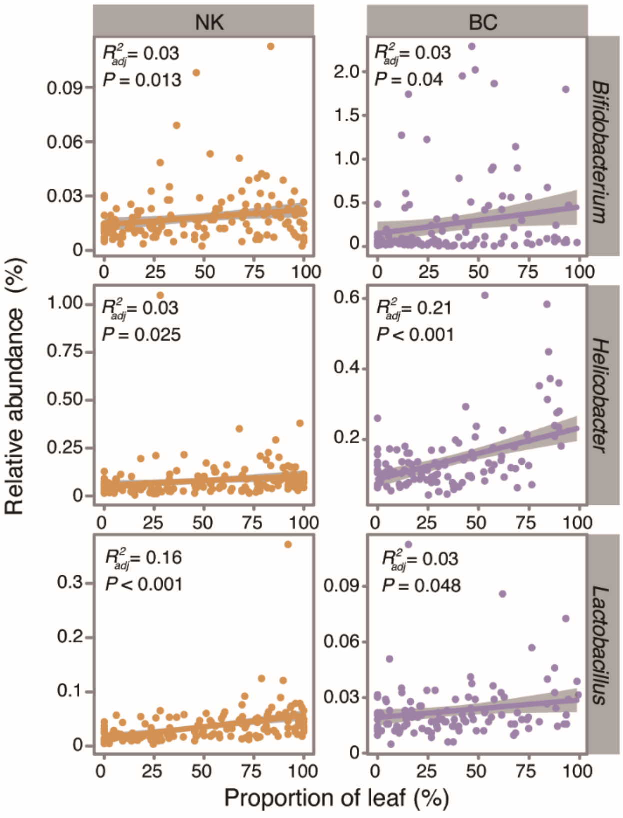

Supplementary Figure 13 | Correlations between the health-related bacterial taxa (family-level) and the diet. The lines and confidence intervals were obtained from the linear regression. Grey shadow represented the 95% confidence interval. Colors denoted different social groups (orange, NK; purple, BC). The adjusted R^2 values for linear regression were presented. The *p*-values were obtained from linear regression modeling.

Supplementary Table 1 The abundance of the core ZOTUs appears in over 90% of the samples.

Supplementary Table 2 SIMPER analysis results display the top 10 ZOTUs responsible for the dissimilarity between two social groups.

Supplementary Table 3 Information of diet-responsive ZOTUs.

Supplementary Table 4 The enrichment result of MAGs in different diet

Supplementary Table 5 The summarized taxonomic and metabolic potentials of the enriched MAGs in high-leaf periods.

Supplementary Table 6 Validation of predictive models for relative abundances of dominant microbial taxa.

Supplementary Table 7 Metadata of the gut microbiome samples of gibbons.

Supplementary Table 8 Taxonomic classification of ZOTUs.

Supplementary Table 9 The abundance of selected KEGG pathways in Fig 5 and Supplementary Figure 10.

Supplementary Table 10 The abundance of GH families associated with carbohydrate degradation.
